# The Comparatively Proteomic Analysis in Response to Cold Stress in Cassava Plantlets

**DOI:** 10.1007/s11105-016-0987-x

**Published:** 2016-05-06

**Authors:** Feifei An, Genghu Li, Qing X. Li, Kaimian Li, Luiz J. C. B. Carvalho, Wenjun Ou, Songbi Chen

**Affiliations:** 1 Tropical Crops Genetic Resources Institute, Chinese Academy of Tropical Agricultural Sciences/Key Laboratory of Ministry of Agriculture for Germplasm Resources Conservation and Utilization of Cassava, Danzhou, 571737 China; 2 Department of Molecular Biosciences and Bioengineering, University of Hawaii at Manoa, Manoa, HI USA; 3 Genetic Resources and Biotechnology, Embrapa, Brazil

**Keywords:** Cassava plantlets, Comparative proteome, Cold stress, Differential proteins

## Abstract

Cassava (*Manihot esculenta* Crantz) is a tropical root crop and sensitive to low temperature. However, it is poorly to know how cassava can modify its metabolism and growth to adapt to cold stress. An investigation aimed at a better understanding of cold-tolerant mechanism of cassava plantlets was carried out with the approaches of physiology and proteomics in the present study. The principal component analysis of seven physiological characteristics showed that electrolyte leakage (EL), chlorophyll content, and malondialdehyde (MDA) may be the most important physiological indexes for determining cold-resistant abilities of cassava. The genome-wide proteomic analysis showed that 20 differential proteins had the same patterns in the apical expanded leaves of cassava SC8 and Col1046. They were mainly related to photosynthesis, carbon metabolism and energy metabolism, defense, protein synthesis, amino acid metabolism, signal transduction, structure, detoxifying and antioxidant, chaperones, and DNA-binding proteins, in which 40 % were related with photosynthesis. The remarkable variation in photosynthetic activity and expression level of peroxiredoxin is closely linked with expression levels of proteomic profiles. Moreover, analysis of differentially expressed proteins under cold stress is an important step toward further elucidation of mechanisms of cold stress resistance.

**Electronic supplementary material:**

The online version of this article (doi:10.1007/s11105-016-0987-x) contains supplementary material, which is available to authorized users.

## Introduction

Being sessile, plants mainly depend on physiological and metabolic adaptations to obtain the phenotypic flexibility required to withstand the adverse biotic and abiotic growth conditions (Sergeant and Renaut 2010), such as drought, low temperature, and salinity. Low temperature has a great impact on plant productivity, mostly because it significantly alters plant metabolism and physiology (Timperio et al. 2008). Cassava (*Manihot esculenta* Crantz) is a staple food for more than 800 million people in the world (Lebot 2008). As a tropical root crop, cassava is sensitive to low temperature (Huang et al. 2005). It can modify its metabolism and growth to adapt to cold stress by reprogramming gene expression to increase the ability to withstand oxidative stress and synthesis of cold-induced proteins during cold acclimation (Kjellsen et al. 2010).

Plants have evolved elaborating mechanisms that allow them to perceive the external signals and to manifest adaptive responses with appropriate physiological changes (Hashimoto and Komatsu 2007). Under cold stress, the plasma membrane undergoes phase transition, from the liquid crystalline to a rigid gel phase (Lyons 1973). The capacity for O_2_ uptake and delivery was reduced, and excess O_2_ in the metabolic process was converted into reactive oxygen species (ROS) (Van Breusegem et al. 1999). At high concentration, ROS cause damage to cell structures and biomolecules; thus, plant cells trigger antioxidant networks to scavenge excessively produced ROS (Koehler et al. 2012; Raimbault et al. 2011; Haghjou et al. 2009). In addition, the compatible osmolytes, such as proline, betaine, and soluble sugars, were increased under cold stress (Hare et al. 1998; Grimaud et al. 2013). The prolonged exposure to low temperature will also decrease the chlorophyll content of plants (Liu et al. 2012; Zhou et al. 2012a, b). Response to cold stress for 4 h in cassava showed that MDA concentrations rapidly decreased to 50 %, but proline dramatically increased and sugar content remained unchanged, but obviously increased after 24 h (An et al. 2012).

Using plant transformation methods, many plants could be improved their abilities to adapt the cold stresses, such as transforming *AtproDH* and *CBF3* genes in *Arabidopsis thaliana* (Nanjo et al. 1999; Gilmour et al. 1998), *ZmMPK4* and *MfGolS1* genes in tobacco (Zhou et al. 2012a, b; Zhuo et al. 2013), *RdreB1BI* gene in strawberries (Gu et al. 2013), *AtDREB1A/CBF3* gene in *Lolium perenne* (Li et al. 2011), and *P5CS* gene in larch (Gleeson et al. 2005). With the recent completion of the cassava genome sequence, many genes in cassava associated with cold tolerance were identified (Vergnolle et al. 2005; Rabbani et al. 2003; Wang et al. 2014). Coupled expression of Cu/Zn-superoxide dismutase (SOD) and catalase was presented in cassava by transforming both *Cu/Zn-SOD* and *CAT* genes to improve tolerance against cold and drought stresses (Xu et al. 2013). Transformation of *CBF3* gene in cassava could enhance the cold tolerance (Liu et al. 2011), and the expression of native cytosolic transformed *SOD* and ascorbate peroxidase (*APX*) genes simultaneously activated the antioxidative defense mechanisms via cyclic ROS scavenging, thereby improved cassava tolerance to cold stress (Xu et al. 2014). At RNA level, a total of 508 transcripts in cassava were identified as early cold-induced genes of which 319 sequences had functional descriptions aligned with *Arabidopsis* proteins (An et al. 2012).

The whole genome and transcriptome may provide comprehensive information about the physiological state of cassava plant and its organisms in a particular condition; however, the levels of global transcripts are not strictly correlated to the levels of the translated proteins (Ideker et al. 2001; Hajduch et al. 2010). In addition, many crucial post-translational modifications may not be screened by transcript analysis (Balbuena et al. 2011). Proteomic analysis has the potential to provide a broad view of plant responses to stress at the level of proteins (Lehesranta et al. 2005). Proteome analyses of cold responses have been carried out in different plant organisms, such as *Arabidopsis* (Amme et al. 2006; Fanucchi et al. 2012), wheat (Rinalducci et al. 2012), rice (Neilson et al. 2011; Cui et al. 2005), pea (Dumont et al. 2011), strawberry (Gu et al. 2013), sunflower (Balbuena et al. 2011), potato (Folgado et al. 2013), tomato (Sanchez-Bel et al. 2012), and soybean (Swigonska and Weidner 2013). However, little is known regarding the effect of cold treatment on the cassava global protein networks that underlie the key physiological processes (Sheffield et al. 2006; Li et al. 2010; Carvalho et al. 2012; An et al. 2014).

In this study, physiological and biochemical characteristics of apical shoots from cassava subjected to low temperature were analyzed. The changes of proteome pattern were studied with 2-DE in combination with MALDI-TOF-MS/MS. All differentially expressed proteins were clustered into cohesive groups based on their biological functions. The network of protein-protein was established to describe the protein interaction against stresses. Our data would be useful to further elucidate the mechanisms of cassava tolerance to cold stress and provide a new clue to cassava breeding.

## Materials and Methods

### Plant Material, Growth Condition, and Cold Treatments

In vitro plantlets of cassava cultivars SC8 and Col1046 were grown at 25 °C under a 16-h photoperiod (100–120 μmol m^−2^ s^−1^) for 40 days in tissue culture room. Plantlets with a uniform growth were transferred to a chamber at 25 °C under a 16-h photoperiod for 1 day, and then, the chamber temperature was dropped from 25 to 5 °C by 0.03 °C/min for cold treatment under weak light (approximately 54 μmol m^−2^ s^−1^). The apical expanded leaves exposed to 5 °C for 0, 3, 7, 10, and 15 days were harvested and then frozen in liquid nitrogen and held at −80 °C. Zero day was used as control. Three biological replicates were conducted in the present study.

### Physiological Analyses of Cold-Treated Cassava Plants

To analyze the physiological changes of cassava under cold treatment, chlorophyll content, electrolyte leakage (EL), free proline content, malondialdehyde (MDA) content, and soluble sugar content were measured for cassava cultivars SC8 and Col1046. Chlorophyll was isolated from the apical expanded leaves according to the procedure of Hu et al. (2005). Chlorophyll content was calculated as described by Porra et al. (1989). EL was measured as described by Cao et al. (2007) with minor modifications. Proline content was measured according to the sulfosalicylic acid-acid ninhydrin method (Bates et al. 1973) with slight modifications. The MDA content was determined by the thiobarbituric acid (TBA) reaction with minor modifications (Dhindsa et al. 1981). Soluble sugars were extracted from the leaf tissues which were treated by cold stress according to the procedure of An et al. (2012).

### Detection of SOD and POD Activities

The fully apical expanded leaves (1 g) in cassava cultivars SC8 and Col1046 were homogenized in 5 mL of 10 mM potassium phosphate buffer (pH7.0) containing 4 % (*w*/*v*) polyvinylpyrrolidone. The homogenate was centrifuged at 10,000 rpm for 15 min, and then, the supernatant was used as the enzyme extract. All steps were carried out at 4 °C. SOD activity assay was based on the method described by Beaucham and Fridovic (1971), which measures the inhibition of the photochemical reduction of nitro blue tetrazolium (NBT) at 560 nm. Three milliliters of reaction mixture contained 50 mM phosphate buffer (pH 7.8), 0.1 mM EDTA, 13 mM methionine, 75 μM NBT, 16.7 μM riboflavin, and 300 μL of enzyme extract. Peroxidase (POD) activity assay was based on the method described by Quintanilla-Guerrero et al. (2008).

### Recovery of Plant Material After Cold Treatments

In vitro plants of cassava cultivars (SC8 and Col1046) treated at 5 °C for 15 days were used as the materials for recovery experiment. The chamber temperature was increased from 5 to 25 °C by 0.03 °C/min with a 16-h photoperiod (100–120 μmol m^−2^ s^−1^).

#### Protein Extraction, 2-DE Separation, and Protein Identification

Proteins from the apical expanded leaves of SC8 and Col1046 plantlets exposed to 5 °C for 10 days were extracted with phenol according to Chen et al. (2009). Plantlets of two genotypes grown in tissue culture room were used as the control. 2-DE protein separation was conducted as previously described in An et al. (2014). Three independent biological replications were carried out in the present study. Gel matching for protein quantification was performed by Delta2D software (Decodon GmbH, Greifswald, Germany), and spot pairs were confirmed visually. The significance of differences was determined by Scheffe’s test at *P* < 0.05. The abundance of each protein spot was estimated by the percentage volume (% vol). Tryptic in-gel digestion and protein identification were performed by the methods reported by An et al. (2014).

### Generation of Protein Interaction Networks

The differential expressed proteins identified from cassava cultivars SC8 and Col1046 were used to generate a wider protein interaction map by employing a Pathway Studio software program (www.ariadnegenomics.com) (Chen et al. 2009) and KEGG pathway software (http://www.genome.jp/kegg/pathway.html).

### Chlorophyll Fluorescence Measurement

The effective PS II quantum yield (ΦPSII) of SC8 and Col1046 leaves under cold stress for 10 days was carried out with the Maxi-version of the Imaging-PAM and the software Imaging WIN version 2.39 (both Heinz Walz GmbH, Effeltrich, Germany) according to Oxborough (2004). Cassava leaves exposed to room temperature were used as the control. An integrated CCD camera enables view and records highly resolved digital images of the emitted fluorescence, and the total imaging area was 10 × 13 cm. Plants were adjusted in the dark for 20 min prior to measurement, and a detached leaf with was clamped onto the holder. For each variant, at least three individual plants were used.

### Western Blot Analyses

Leaves of SC8 and Col1046 under cold stress for 10 days were collected for Western blot analysis. The protein extraction and Western blot were performed according to An et al. (2014). Proteins were detected by immunostaining with anti-Rubisco polyclonal antibody (AS07218) and anti-peroxiredoxin antibody (AS05093) from Agrisera. Western blots were developed according to the method of NBT/BCIP from Roche (11681451001).

### Statistical Analyses

All data are represented as means ± SE from three independent experiments with three replications. Statistical analysis was conducted using ANOVA, which was performed by using SPSS 17.0 to Duncan’s tests. A value of *P* < 0.05 was considered a statistically significant difference.

## Results

### Phenotypic and Physiological Changes with Cold Treatment of Cassava

**Fig. 1:**
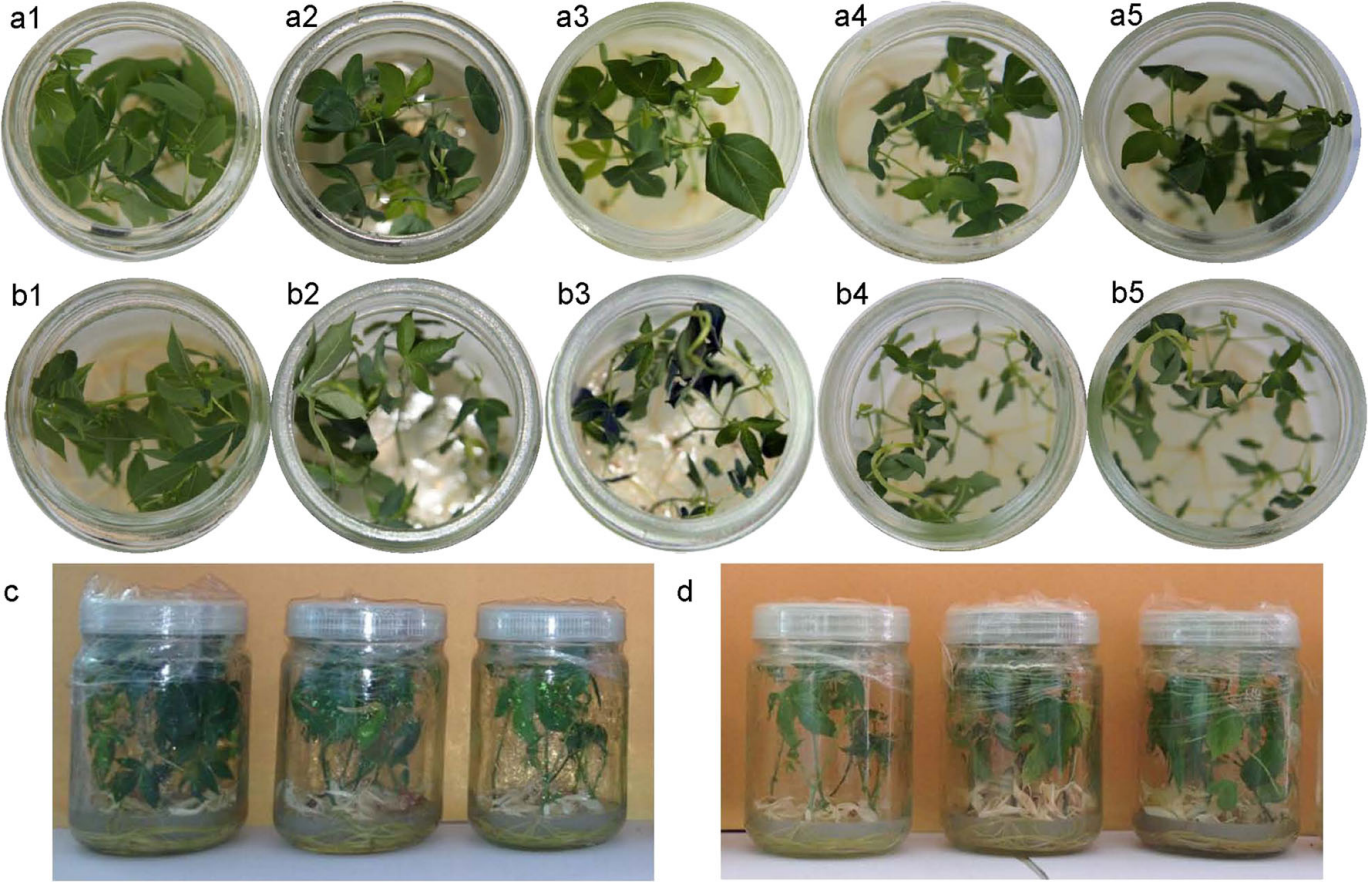
Phenotypic changes in cold-stressed cassava and recovery after cold stress. *a1*–*5*, 40-day-old SC8 subjected to 5 °C for 0, 3, 7, 10, and 15 days in a chamber under light showing phenotypic changes, respectively; *b1*–*5*, 40-day-old Col1046 subjected to 5 °C for 0, 3, 7, 10, and 15 days in a chamber under light showing phenotypic changes, respectively. **c** Cassava SC8 plantlets exposed to 5 °C for 15 days were moved to room temperature for 2 months. **d** Cassava Col1046 plantlets exposed to 5 °C for 15 days were moved to room temperature for 2 months

Compared with in vitro plantlets of cassava cultivars SC8 and Col1046, with vigorous apical buds and fully expanded leaves, in room temperature (Fig. 1 (a1, b1)), the leaves of cassava plantlets exposed to 5 °C for 3 days displayed dehydration and wilting (Fig. 1 (a2, b2)). The phenotypic damages in both genotypes exposed to 5 °C from 7 to 15 days (Fig. 1 (a3–5, b3–5)) showed softening and downward bending of the petioles, loss of strength in the immature stems, and more severe wilting leaves. Especially, the cold treatment caused cassava plantlets of genotype Col1046 more dehydration and wilting than SC8 (Fig. 1 (a2–5, b2–5)). However, the cold-treated plantlets of SC8 (Fig. 1 (a5)) and Col1046 (Fig. 1 (b5)) were transferred into room temperature for 2 months, as shown in Fig. 1c, d; they were recovered, suggesting that cassava phenotypic changes caused by cold treatment were reversible.

**Fig. 2:**
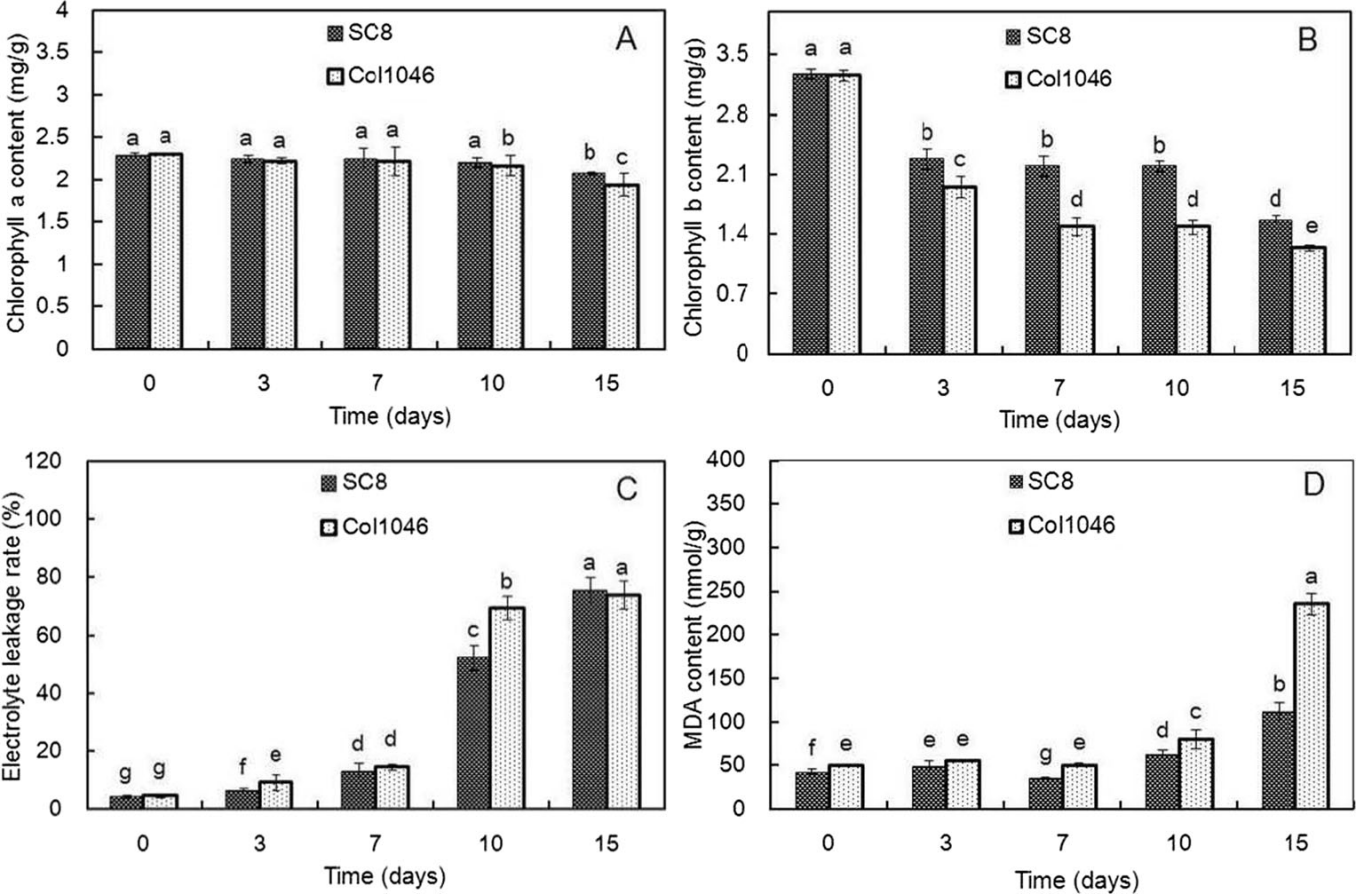
Contents of chlorophyll, EL, and MDA in cold-stressed cassava apical expanded leaves. Chlorophyll a contents (**a**), chlorophyll b contents (**b**), electrolyte leakage (**c**), and MDA (**d**) in SC8 and Col1046 apical leaves exposed to 5 °C for 0, 3, 7, 10, and 15 days. The mean values are calculated from three biological replicates; the *error bars* represent the standard error of the mean

**Fig. 3:**
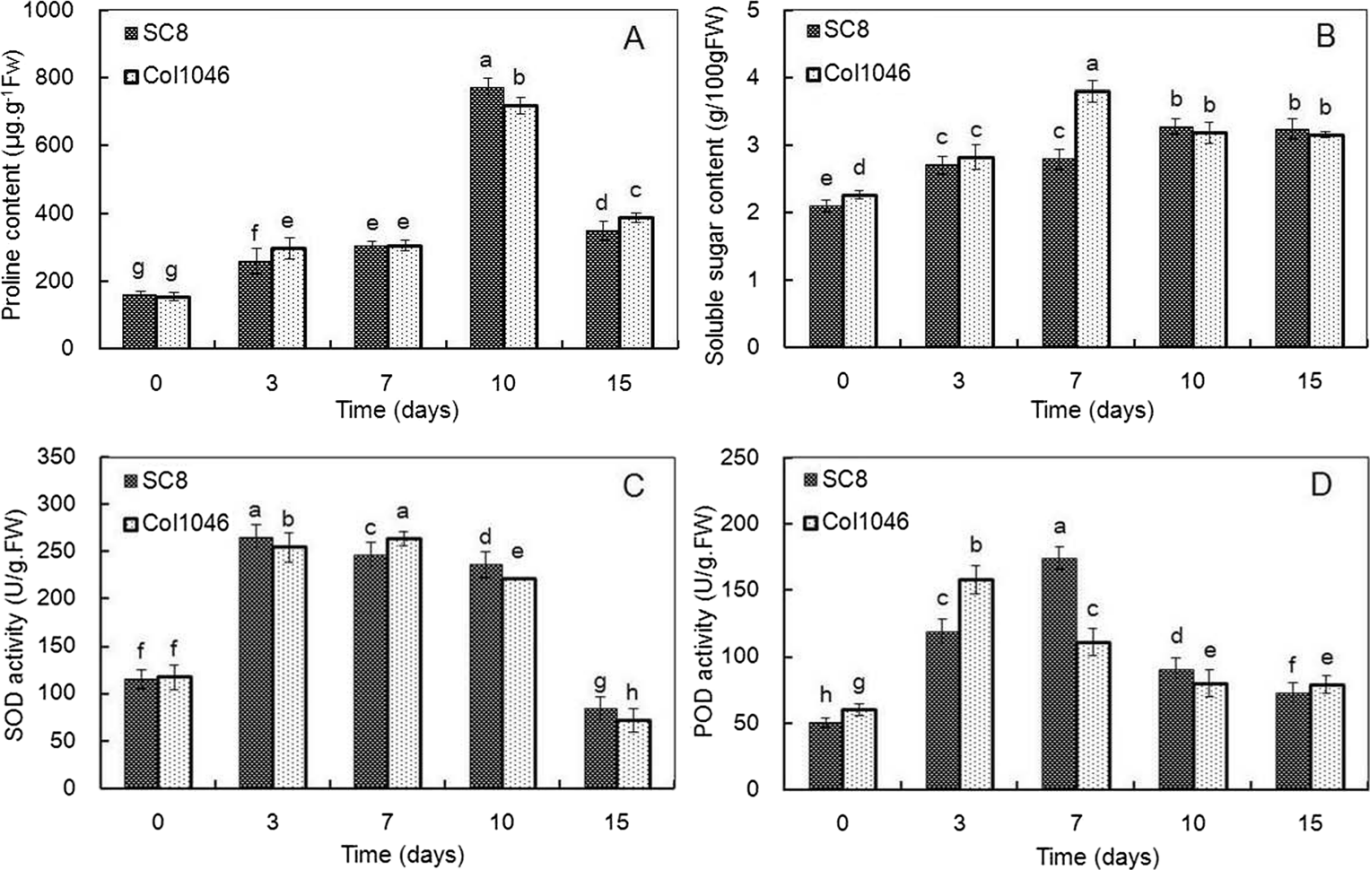
Content proline and soluble sugar and activities of SOD and POD in cold-stressed cassava apical expanded leaves. Proline contents (**a**), soluble sugar contents (**b**), SOD activities (**c**), and POD activities (**d**) in SC8 and Col1046 apical leaves exposed to 5 °C for 0, 3, 7, 10, and 15 days. The mean values are calculated from three biological replicates; the *error bars* represent the standard error of the mean

Cold stress also leads to obviously physiological changes in cassava. As an important element in photosynthesis, chlorophyll plays a key role in the absorption and conversion of light energy (Wettstein et al. 1995). Chlorophyll a content of SC8 and Col1046 exposed to 5 °C for 7 days did not change and showed a slow decrease from 10 to 15 days under cold stress. On the contrary, the content of chlorophyll b of both cassava genotypes decreased rapidly. The decrease levels of chlorophyll a and b contents in Col1046 were more than SC8 (Fig. 2a, b). EL and MDA content of SC8 and Col1046 were significantly enhanced with the extension of the cold stress time (Fig. 2c, d). The results showed that the increased levels of EL and MDA content in Col1046 were more than SC8, suggesting that cell membrane of Col1046 was more sensitive to continuously low temperature than SC8 (Fig. 2c). The significant effects of low temperature on MDA in Col1046 occurred at the exposure to 5 °C from 10 to 15 days (Fig. 2d). The contents of free proline and soluble sugar in two genotypes were gradually increased until reaching the maximum level and then starting to decrease (Fig. 3a, b). The two cassava genotypes had a maximum value of free proline content at 10-day cold stress. SC8 had a maximum value of soluble sugar content at 7-day cold stress; however, Col1046 had a maximum value of soluble sugar content at 10-day cold stress. SOD activities of two genotypes significantly increased under cold stress from 3 to 10 days and then significantly decreased at 15-day cold stress (Fig. 3c). SC8 had the highest SOD activity at 3-day cold stress, but Col1046 had the highest SOD activity at 7-day cold stress (Fig. 3c). Under cold stress, POD activity of two genotypes significantly increased to a maximum level and then started to decrease during 15 days. SC8 had the highest POD activity at 7-day cold stress, but Col1046 had the highest SOD activity at 3-day cold stress (Fig. 3d).

### Correlation and Principal Component Analysis of Physiological Parameters

The correlation analysis of seven physiological and biochemical characteristics (chlorophyll content, EL, MDA content, proline content, soluble sugar content, SOD, and POD) showed that chlorophyll content was negatively correlated with EL, proline content, MDA content, soluble sugar content, and the activity of SOD and POD and significantly negatively with EL, MDA content, and soluble sugar content; EL was significantly positively related with proline and MDA content; SOD activity and POD activity had a significant positive correlation (Table S1). The principal component (PC) analysis showed that EL, chlorophyll content, and MDA may be the most important physiological and biochemical indexes for determining cassava genotypes with cold-resistant abilities (Table S2).

### Protein Profile Responses to Cold Treatment

**Fig. 4:**
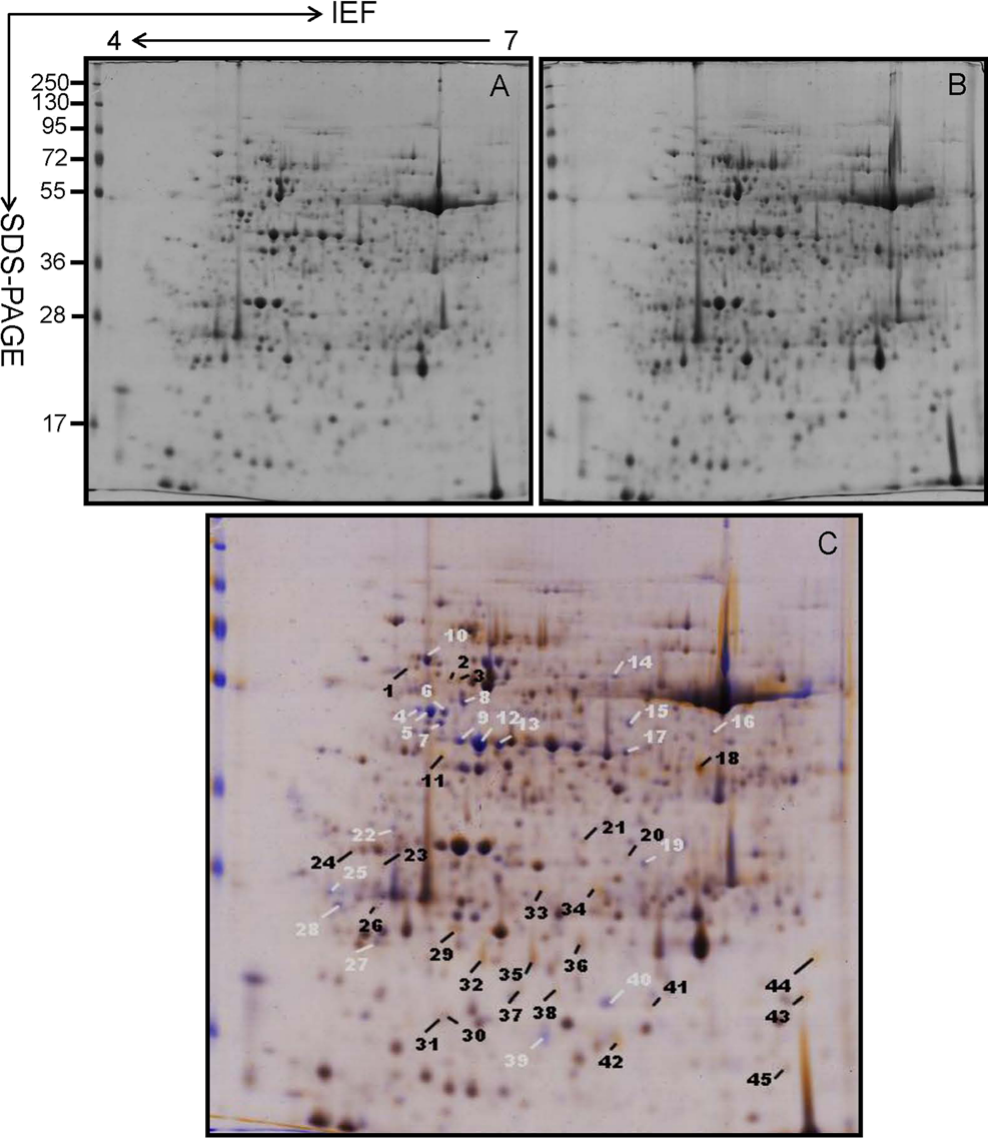
Coomassie-stained 2-D gel protein profiles of SC8 apical expanded leaves: **a** 5 °C for 0 day, **b** 5 °C for 10 days, and **c** wrapped 2-DE map from 5 °C for 0 day and 5 °C for 10 days. The *white* and *black arrows* in **c** indicate proteins that showed detectable changes (>2.0-fold the normalized volume) in abundance compared with those seen in 5 °C for 0 day; *white arrows* indicate a down-regulated match, and *black arrows* indicate an up-regulated match

**Fig. 5:**
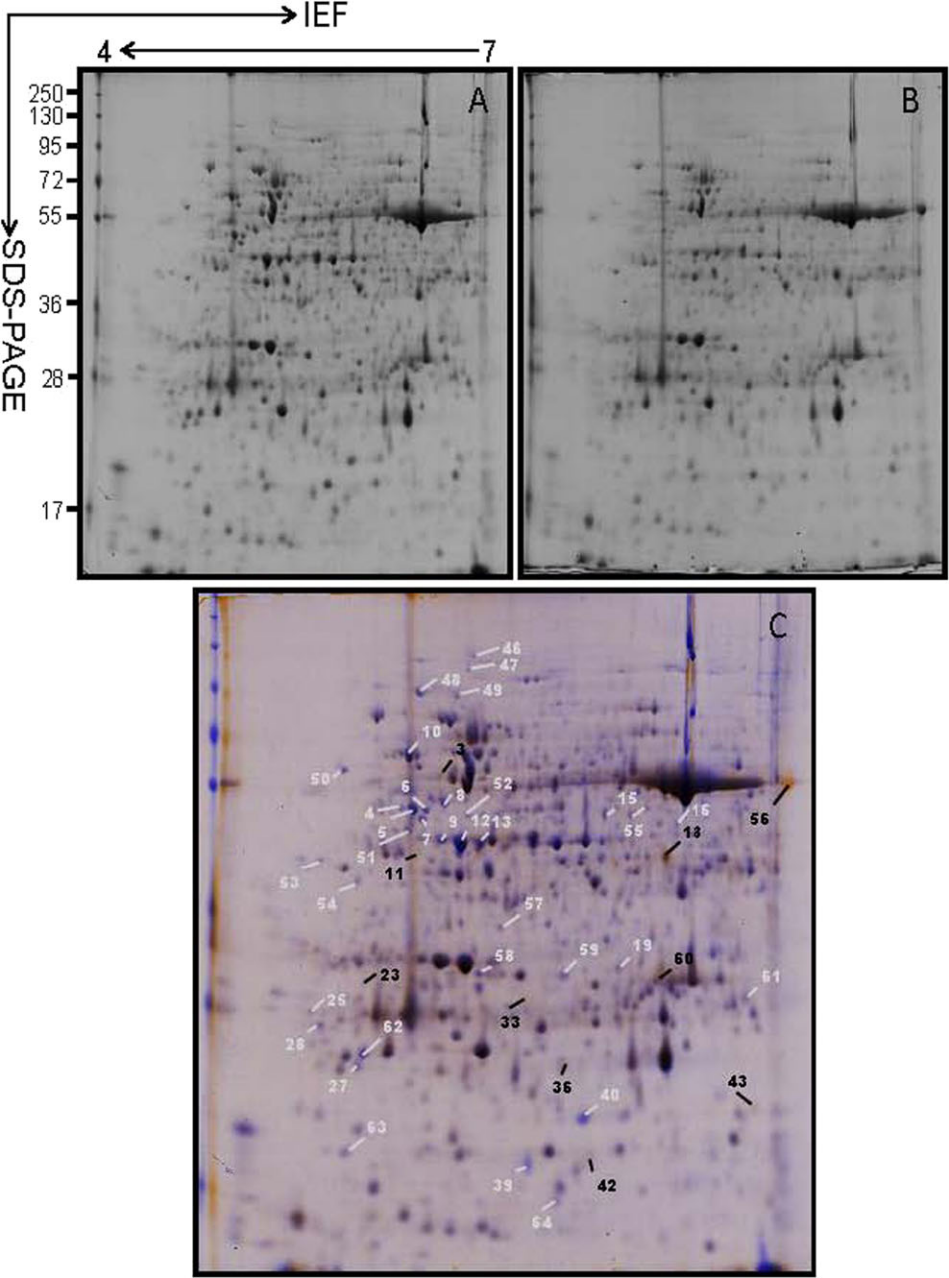
Coomassie-stained 2-D gel protein profiles of Col1046 apical expanded leaves: **a** 5 °C for 0 day, **b** 5 °C for 10 days, and **c** wrapped 2-DE map from 5 °C for 0 day and 5 °C for 10 days. The *white* and *black arrows* in **c** indicate proteins that showed detectable changes (>2.0-fold the normalized volume) in abundance compared with those seen in 5 °C for 0 day; *white arrows* indicate a down-regulated match, and *black arrows* indicate an up-regulated match

Proteins from apical expanded leaves of SC8 and Col1046 were purified from 2D-PAGE gels (Figs. 4a, b and 5a). In total, ∼500 protein spots were detected by digital image analysis, and at least 300 spots gave reproducible staining patterns for all samples as judged by spot intensity ranking. Using a spot-to-spot comparison and statistical analysis, a total of 45 stained spots (Fig. 4c) in SC8 and 44 stained spots (Fig. 5c) in Col1046 were found to have significant changes (*P* < 0.05) with greater than ±2.0-fold altered intensity in all three replicate gels. In SC8, the expressions of 20 spots (spots 4, 5, 6, 7, 8, 9, 10, 12, 13, 14, 15, 16, 17, 19, 22, 25, 27, 28, 39, and 40) were down-regulated and the remainders were up-regulated (Fig. 4c). The expression of ten spots (spots 3, 11, 18, 23, 33, 36, 42, 43, 56, and 60) in Col1046 was up-regulated, and the remainders were down-regulated (Fig. 5c).

### Functional Grouping of Identified Proteins

**Fig. 6:**
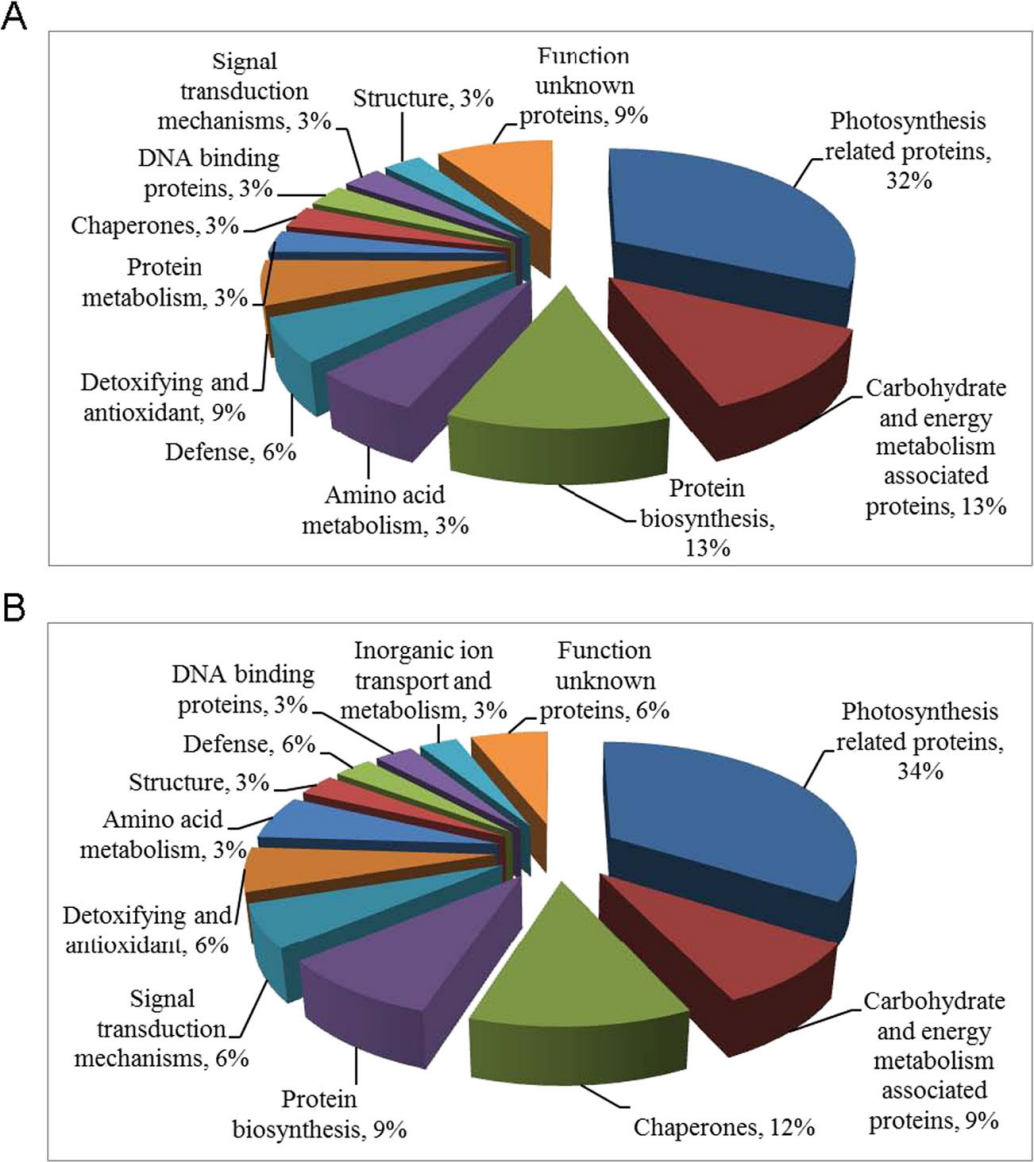
Functional categories of differential proteins. **a** Functional categories of differential proteins in SC8 apical expanded leaves exposed to 5 °C for 10 days compared with 5 °C for 0 day. **b** Functional categories of differential proteins in Col1046 apical expanded leaves exposed to 5 °C for 10 days compared with 5 °C for 0 day. Functional categorization was performed according to MIPS database (http://mips.gsf.de)

Forty-five spots in SC8 and 44 spots in Col1046 with differential expression were isolated from 2-DE gels and identified with MALDI-TOF-MS/MS, of which 32 and 33 protein spots were identified, respectively. In SC8, 17 identified proteins were up-regulated and 15 were down-regulated response to cold stress. Functions of 32 differentially expressed proteins were annotated via the survey of gene banks (Table 1, Fig. 6a). Ten proteins (32 %) are associated with photosynthesis metabolism, of which three proteins (spots 20, 36, 47) were down-regulated. Four up-regulated proteins (13 %) were associated with carbohydrate and energy metabolism, and four proteins (13 %) were involved in protein biosynthesis including one protein (spot 28), which was down-regulated. Two proteins (6 %) in defense system and spot 19 were down-regulated, three proteins (9 %) in detoxifying and antioxidant and spot 27 was down-regulated, one down-regulated protein (3 %) in amino acid metabolism, one up-regulated protein (3 %) with signal transduction, one up-regulated protein (3 %) with structure, one up-regulated protein (3 %) with protein metabolism, one down-regulated protein (3 %) with chaperones, and one down-regulated protein (3 %) with DNA binding. Other three were function-unknown proteins, including two down-regulated proteins.

**Table 1:** Identification of differential expressed proteins in cassava cultivar SC8 tissue culture plantlet apical shoots under low temperature stresses

Spot number^a^	Identification	GI no.^b^	pI/Mw (kDa)	Score^c^	Fold changes^d^ (mean ± SE)
Photosynthesis-related proteins (10)					
4	Putative Rubisco activase protein-*Z. hybrid cultivar*	47176692	5.08/27.69	71	3.03 ± 0.08 (−)
5	Putative Rubisco activase protein-*Z. hybrid cultivar*	47176692	5.08/27.69	90	4.41 ± 0.18 (−)
6	Putative Rubisco activase protein-*Z. hybrid cultivar*	47176692	5.08/27.69	86	2.64 ± 0.07 (−)
7	Ribulose bisphosphate carboxylase activase-*N. tabacum*	19988	4.83/22.98	84	3.73 ± 0.14 (−)
9	Putative Rubisco activase protein-*Z. hybrid cultivar*	47176692	5.08/27.69	88	2.52 ± 0.08 (−)
12	Ribulose bisphosphate carboxylase activase-*N. tabacum*	19988	4.83/22.98	116	5.51 ± 0.24 (−)
13	Ribulose bisphosphate carboxylase activase- *N. tabacum*	19988	4.83/22.98	98	3.17 ± 0.17 (−)
20	Ribulose bisphosphate carboxylase activase small chain SU26,chloroplastic	132094	8.62/19.81	59	2.10 ± 0.07 (+)
36	Oxygen-evolving enhancer protein 2,chloroplastic	131390	8.28/28.05	47	2.28 ± 0.12 (+)
41	Ribulose 1,5-bisphosphate carboxylase small chain precursor-*M. esculenta*	6272551	8.33/20.41	81	2.58 ± 0.12 (+)
Carbohydrate and energy metabolism-associated proteins (4)					
2	ATP synthase CF1 beta subunit-*B. napus*	262400757	5.14/53.75	92	3.50 ± 0.15 (+)
3	ATP synthase subunit beta, chloroplastic	114557	5.15/53.47	103	2.69 ± 0.26 (+)
18	Phosphoglycerate kinase-*A. thaliana*	1022805	4.93/41.91	111	3.92 ± 0.16 (+)
21	Carbohydrate kinase FGGY-*K. flavida DSM 17836*	284028646	5.80/47.83	75	2.07 ± 0.01 (+)
Protein biosynthesis (4)					
1	Integrase catalytic region-*Rhizobium sp. PDO1-076*	375054862	9.71/59.54	73	2.21 ± 0.05 (+)
11	Molybdenum cofactor biosynthesis protein A	59798352	8.38/35.62	80	∞ (+)
28	Proteasome subunit alpha type-5	12229923	4.70/25.98	136	2.67 ± 0.10 (−)
44	30S ribosomal protein S3	61215424	10.28/29.61	71	∞ (+)
Defense (2)					
19	CDSP32 Protein (Chloroplast drought-induced stress protein of 32kDa)-*S. tuberosum*	2582822	8.07/33.46	101	2.38 ± 0.13 (−)
43	Metacaspase-9-*A. thaliana*	75263209	5.81/35.51	81	∞ (+)
Detoxifying and antioxidant (3)					
27	2-Cys peroxiredoxin-like protein-*H. orientalis*	47027073	4.93/21.86	109	2.30 ± 0.11 (−)
29	Putative glutathione S-transferase protein-*P. tunicata D2*	88857201	5.88/24.25	86	3.28 ± 0.23 (+)
34	Ascorbate peroxidase APX3-*M. esculenta*	62526589	5.31/27.67	76	7.66 ± 0.52 (+)
Amino acid metabolism (1)					
39	5-Methyltetrahydropteroyltriglutamate/homocysteine S-methyltransferase-*Metallosphaera sedula DSM 5348*	146304995	9.54/ 36.30	88	23.6 ± 1.23 (−)
Chaperones (1)					
10	Rubisco subunit binding-protein alpha subunit-*R. communis*	255587664	5.25/53.20	177	2.13 ± 0.06 (−)
DNA binding proteins (1)					
40	DNA-binding protein-*Z. mays*	397396	5.87/18.29	59	3.60 ± 0.31 (−)
Protein metabolism (1)					
26	ATP-dependent Clp protease proteolytic subunit-*A. thaliana*	21593305	5.37/31.51	58	2.17 ± 0.04 (+)
Signal transduction mechanisms (1)					
23	14-3-3-like protein	12229593	4.79/29.25	84	∞ (+)
Structure (1)					
42	Similar to actin-binding protein-*A. thaliana*	3047107	5.12/15.70	44	∞ (+)
Function unknown proteins (3)					
17	Hypothetical protein-*P. anserina S mat*	171680357	9.61/ 31.52	85	2.09 ± 0.02 (−)
22	Hypothetical protein-*V. vinifera*	147800453	5.43/49.83	68	2.58 ± 0.13 (−)
31	Hypothetical protein-*E. siliculosus*	298710091	6.15/18.19	97	2.06 ± 0.02 (+)
The total protein number		32			

After gel analysis and manual editing with Delta2D software, the spots showing altered expression (>2.0-fold the normalized volume) were counted. Each value represents the mean ± SE of triplicates. Protein spots whose abundance increased (+) or decreased (−) after polyploidy are shown

^a^The numbering corresponds to the 2-DE gel in Fig. 4c

^b^GI number

^c^Probability-based molecular weight search (MOWSE) scores

^d^Expression change level after cold stress

In Col1046, nine identified proteins were up-regulated and 24 were down-regulated response to cold stress. Functions of 33 differentially expressed proteins were annotated via the survey of gene banks (Table 2, Fig. 6b). Eleven proteins (34 %) are associated with photosynthesis metabolism, of which two proteins (spots 36, 60) were up-regulated. Four down-regulated proteins (12 %) were chaperones, three proteins (9 %) were associated with carbohydrate and energy metabolism including one down-regulated protein (spot 52), and three proteins (9 %) were involved in protein biosynthesis including one protein (spot 11), which was up-regulated. Two proteins (6 %) were involved in defense system, two down-regulated proteins (6 %) in detoxifying and antioxidant, and one down-regulated protein (3 %) in amino acid metabolism. Two up-regulated proteins (6 %) were associated with signal transduction, one up-regulated protein (3 %) with structure, one down-regulated protein (3 %) with inorganic ion transport and metabolism, and one down-regulated protein (3 %) with DNA binding. Other two down-regulated spots were function-unknown proteins.

**Table 2:** Identification of differential expressed proteins in cassava cultivar Col1046 tissue culture plantlet apical shoots under low temperature stresses

Spot number^a^	Identification	GI no.^b^	pI/Mw (kDa)	Score^c^	Fold changes^d^ (mean ± SE)
Photosynthesis-related proteins (11)					
4	Putative Rubisco activase protein-*Z. hybrid cultivar*	47176692	5.08/27.69	71	4.67 ± 0.34 (−)
5	Putative Rubisco activase protein-*Z. hybrid cultivar*	47176692	5.08/27.69	90	2.43 ± 0.23 (−)
6	Putative Rubisco activase protein*-Z. hybrid cultivar*	47176692	5.08/27.69	86	2.49 ± 0.12 (−)
7	Ribulose bisphosphate carboxylase activase-*N. tabacum*	19988	4.83/22.98	84	3.76 ± 0.36 (−)
9	Putative Rubisco activase protein-*Z. hybrid cultivar*	47176692	5.08/27.69	88	2.13 ± 0.12 (−)
12	Ribulose bisphosphate carboxylase activase-*N. tabacum*	19988	4.83/22.98	116	2.83 ± 0.43 (−)
13	Ribulose bisphosphate carboxylase activase*-N. tabacum*	19988	4.83/22.98	98	2.93 ± 0.35 (−)
36	Oxygen-evolving enhancer protein 2, chloroplastic	131390	8.28/28.05	47	2.12 ± 0.1 (+)
51	Ribulose bisphosphate carboxylase activase-*N. tabacum*	19988	4.83/22.98	78	2.06 ± 0.02 (−)
60	Carbonic anhydrase, chloroplastic	115472	6.61/34.57	131	2.31 ± 0.21 (+)
61	Carbonic anhydrase, chloroplastic	115472	6.61/34.57	105	3.61 ± 0.25 (−)
Carbohydrate and energy metabolism-associated proteins (3)					
3	ATP synthase subunit beta, chloroplastic	114557	5.15/53.47	103	∞ (+)
18	Phosphoglycerate kinase-*A. thaliana*	1022805	4.93/41.91	111	7.22 ± 0.63 (+)
52	Succinate dehydrogenase-*R. communis*	255579273	6.18/68.51	117	3.24 ± 0.28 (−)
Protein biosynthesis (3)					
11	Molybdenum cofactor biosynthesis protein A	59798352	8.38/35.62	80	∞ (+)
28	Proteasome subunit alpha type-5	122299231	4.70/25.98	136	2.07 ± 0.06 (−)
58	Uncharacterized HTH-type transcriptional regulator	1176726	8.72/21.35	70	2.59 ± 0.03 (−)
Defense (2)					
19	Chloroplast drought-induced stress protein of 32 kDa (CDSP32 protein)-*S. tuberosum*	2582822	8.07/33.46	101	2.65 ± 0.34 (−)
43	Metacaspase-9-*A. thaliana*	75263209	5.81/35.51	81	5.45 ± 0.47 (+)
Detoxifying and antioxidant (2)					
27	2-Cys peroxiredoxin-like protein-*H. orientalis*	47027073	4.93/21.86	109	2.23 ± 0.21 (−)
62	Peroxiredoxin-*P. vulgaris*	11558242	5.18/28.62	123	2.01 ± 0.05 (−)
Amino acid metabolism (1)					
39	5-Methyltetrahydropteroyltriglutamate/homocysteine S-methyltransferase-*Metallosphaera sedula DSM 5348*	146304995	9.54/ 36.30	88	25.74 ± 2.36 (−)
Chaperones (4)					
10	Rubisco subunit binding-protein alpha subunit-*R. communis*	255587664	5.25/53.20	177	2.00 ± 0.02 (−)
57	Shoot1 protein-*G. max*	13650078	5.26/40.24	91	2.07 ± 0.04 (−)
59	Binding/catalytic/coenzyme binding-*A. thaliana*	18404496	8.37/34.88	263	2.19 ± 0.09 (−)
64	HSP19 class II-*Citrus* × *paradisi*	30575570	8.01/11.14	67	2.05 ± 0.06 (−)
DNA binding proteins (1)					
40	DNA-binding protein-*Z. mays*	397396	5.87/18.29	59	6.49 ± 0.56 (−)
Signal transduction mechanisms (2)					
23	14-3-3-like protein	12229593	4.79/29.25	84	34.11 ± 2.56 (+)
56	Adenylate cyclase-like protein-*Thermus thermophilus* HB8	55981228	9.76/95.97	91	18.92 ± 1.54 (+)
Structure (1)					
42	Similar to actin-binding protein-*A. thaliana*	3047107	5.12/15.70	44	3.72 ± 0.56 (+)
Inorganic ion transport and metabolism (1)					
50	Calreticulin-*A. thaliana*	1009712	4.37/46.58	228	2.09 ± 0.02 (−)
Function unknown proteins (2)					
48	Hypothetical protein-*S. moellendorffii*	302784390	8.40/91.35	76	2.90 ± 0.07 (−)
63	Unknown protein-*A. thaliana*	4567284	9.30/25.55	88	2.03 ± 0.03 (−)
The total protein number		33			

After gel analysis and manual editing with Delta2D software, the spots showing altered expression (>2.0-fold the normalized volume) were counted. Each value represents the mean ± SE of triplicates. Protein spots whose abundance increased (+) or decreased (−) after polyploidy are shown

^a^The numbering corresponds to the 2-DE gel in Fig. 5c

^b^GI number

^c^Probability-based molecular weight search (MOWSE) scores

^d^Expression change level after cold stress

**Fig. 7:**
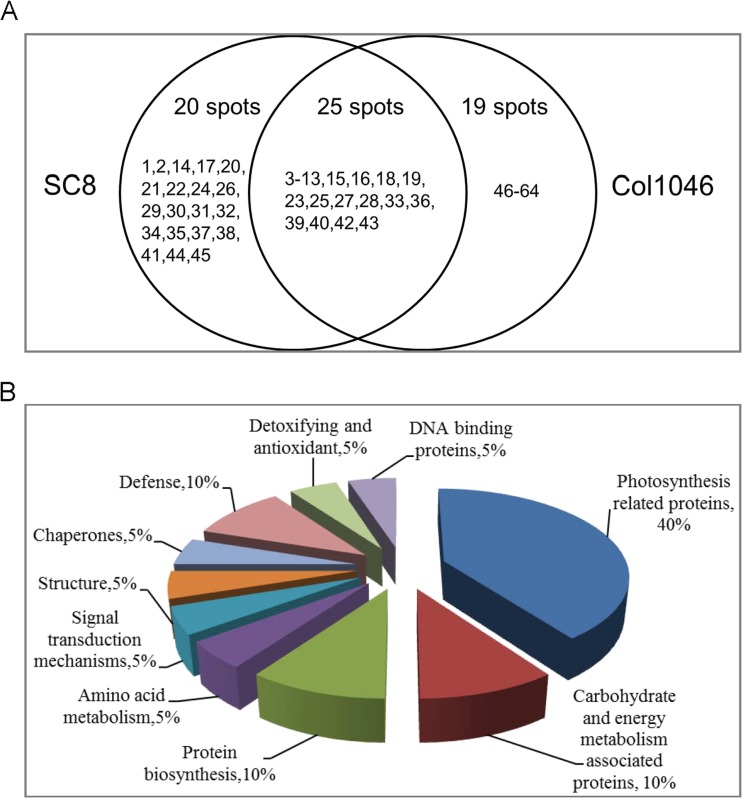
Functional categories of the common differential proteins identified in both SC8 and Col1046 apical expanded leaves exposed to 5 °C for 10 days compared with 5 °C for 0 day. **a** Number of spots altered in expression of SC8 and Col1046. **b** Functional categories of 20 common differential proteins in SC8 and Col1046

As shown in Fig. 7a, 25 differential spots showed the same changes in SC8 and Col1046, of which 20 protein spots were identified and functions were annotated (Fig. 7b). Eight proteins (40 %) are associated with photosynthesis metabolism, of which spot 36 was up-regulated. Two up-regulated proteins (10 %) were associated with carbohydrate and energy metabolism, and two proteins (10 %) were involved in protein biosynthesis including one protein (spot 11), which was up-regulated. Spot 39 (5 %) in amino acid metabolism was down-regulated. One down-regulated protein (5 %) was chaperone, and two proteins (10 %) were in defense system, one down-regulated protein (5 %) in detoxifying and antioxidant, and one down-regulated protein (5 %) with DNA binding. One up-regulated protein (5 %) was with signal transduction and one up-regulated protein (5 %) with structure.

**Fig. 8:**
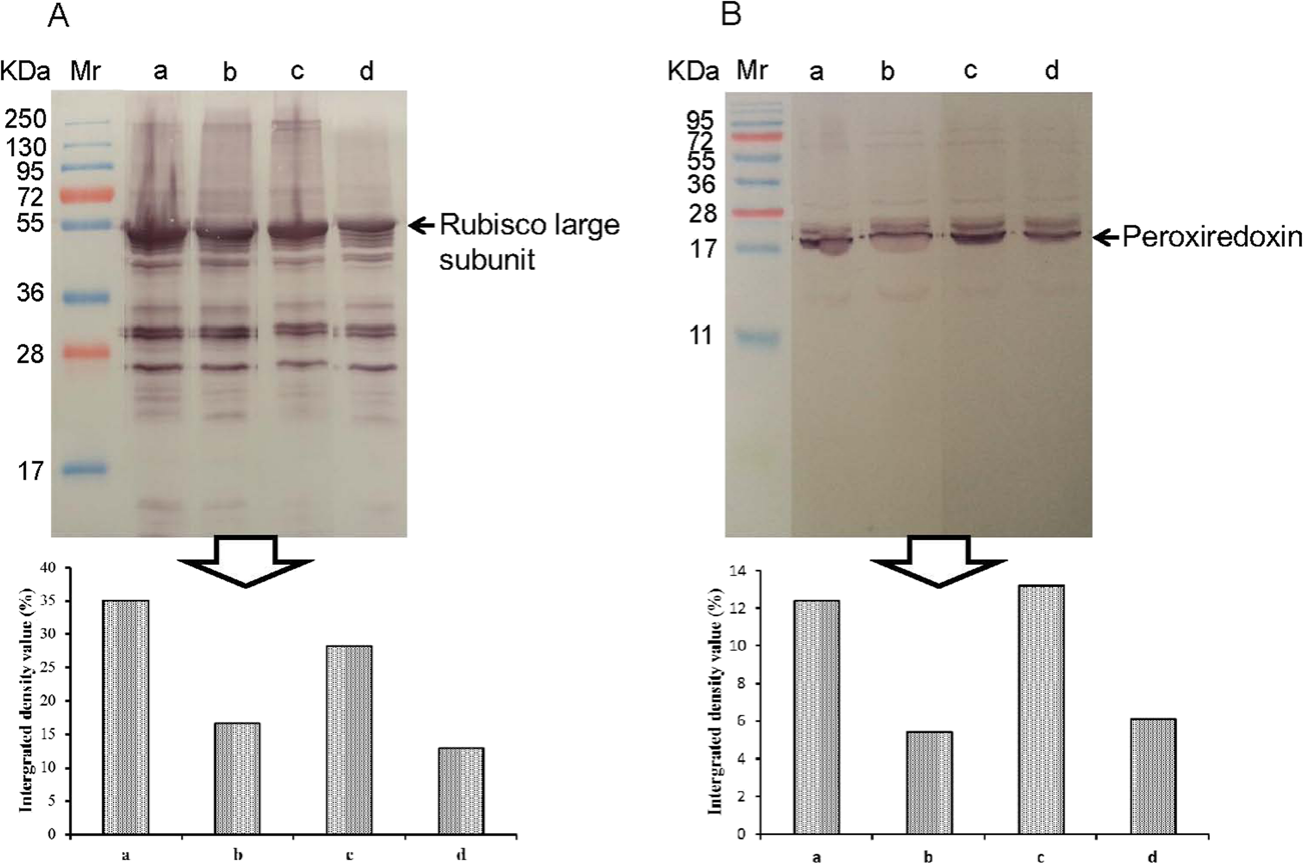
Western blotting of Rubisco and peroxiredoxin. The expressions of Rubisco (**a**) and peroxiredoxin (**b**) in apical expanded leaves of SC8and Col1046 were detected by Western blotting using anti-Rubisco polyclonal antibody (AS07218) and anti-peroxiredoxin antibody (AS05093) from Agrisera, respectively. *Mr*, protein marker; *lines a* and *b*, the expression of Rubisco and peroxiredoxin of SC8 in 5 °C for 0 and 10 days, respectively; *lines c* and *d*, the expression of Rubisco and peroxiredoxin of Co11046 in 5 °C for 0 and 10 days, respectively

To ensure reliability of differential proteins observed on 2-DE gels, the protein expressions of Rubisco and peroxiredoxin in apical expanded leaves of cassava SC8 and Col1046 exposed to cold stress for 10 days were detected by Western blot to validate the proteomic analysis (Fig. 8). The results showed that the expression levels of Rubisco and peroxiredoxin in SC8 and Col1046 were similar with those seen on 2-DE images (Figs. 4 and 5).

### Photosynthetic Activities in Low-Temperature-Treated Cassava

**Fig. 9:**
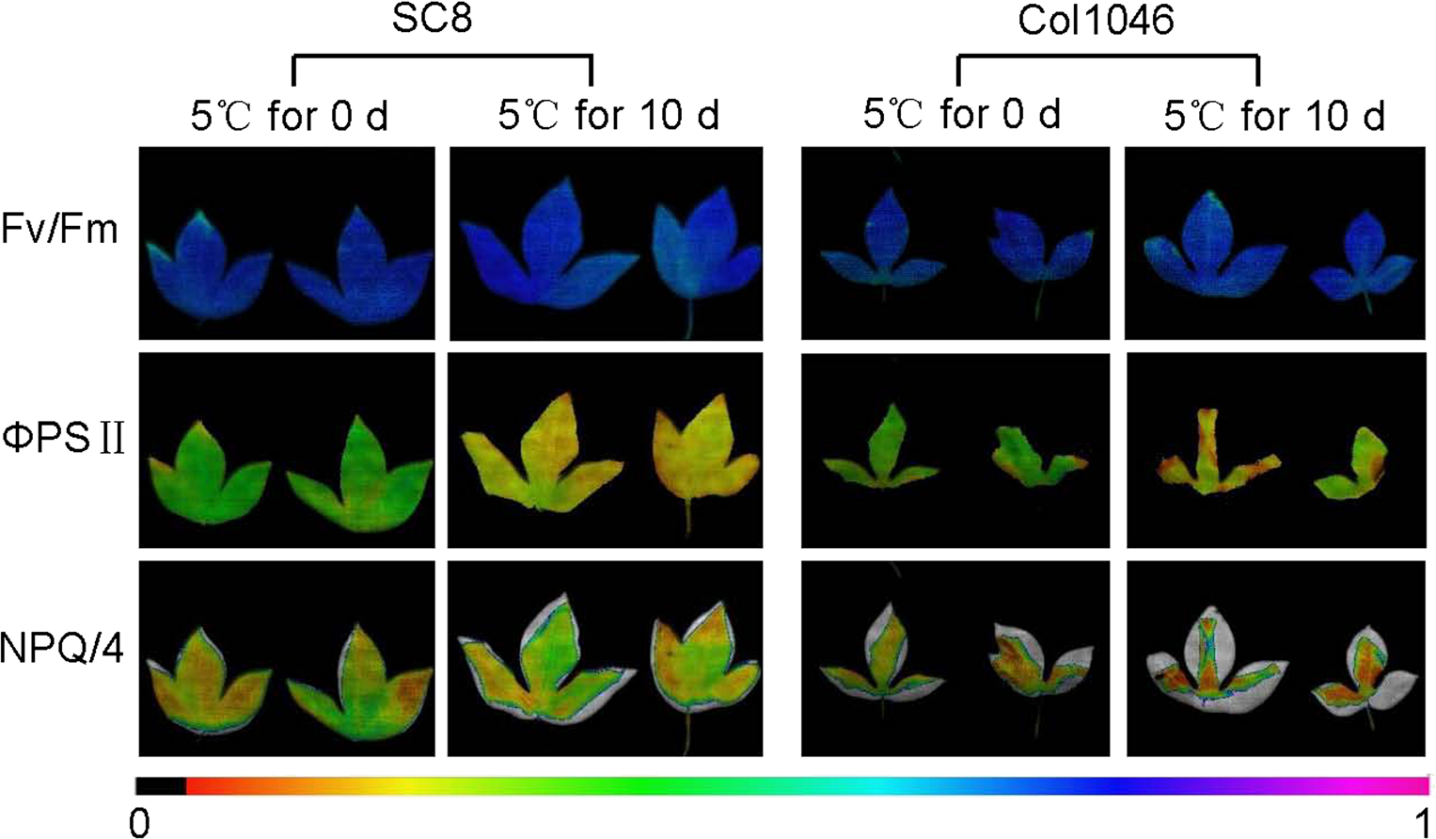
Imaging pulse amplitude modulation of SC8 and Col1046 collected from apical expanded leaves exposed to 5 °C for 0 and 10 days. Parameters were Fv/Fm (maximal photosystem II (PSII) quantum yield), ΦPSII (effective PSII quantum yield), and NPQ/4 (non photochemical quenching). The *color gradient* provided a scale from 0 to 100 % for assessing the magnitude of the parameters

In order to understand the effects of cold stress on the photosynthetic activities of cassava in vitro plants, the photosynthesis parameters Fv/Fm, ΦPSII, NPQ/4, and qN of the 10-day cold stress leaves of SC8 and Col1046 were determined using Imaging-PAM. Figure 9 and Table 3 show that the Fv/Fm and ΦPSII of both genotypes were sensitive to low temperature and decreased significantly; however, NPQ/4 and qN were increased significantly. These data imply down-regulated proteins associated with photosynthesis may result in decrease of photosynthetic activities under cold stress.

**Table 3:** Photosynthetic parameters collected from cassava apical shoots of SC8, Col1046, and treatments

Treatment	Fv/Fm (mean ± SE)	ΦPSII (mean ± SE)	NPQ/4 (mean ± SE)	qN (mean ± SE)
SC8 at 5 °C for 0 day	0.725 ± 0.006 A	0.371 ± 0.016 A	0.167 ± 0.023 B	0.537 ± 0.012 B
SC8 at 5 °C for 10 days	0.684 ± 0.008 B	0.198 ± 0.011 B	0.278 ± 0.015 A	0.675 ± 0.008 A
Col1046 at 5 °C for 0 day	0.718 ± 0.012 A	0.353 ± 0.010 A	0.267 ± 0.015 B	0.646 ± 0.043 B
Col1046 at 5 °C for 10 days	0.675 ± 0.007 B	0.227 ± 0.009 B	0.422 ± 0.013 A	0.782 ± 0.013 A

Values were means ± SE. Different capital letters in the same column indicated statistically significant differences according to Duncan test (*P* < 0.01)

#### Protein Interaction Networks

**Fig. 10:**
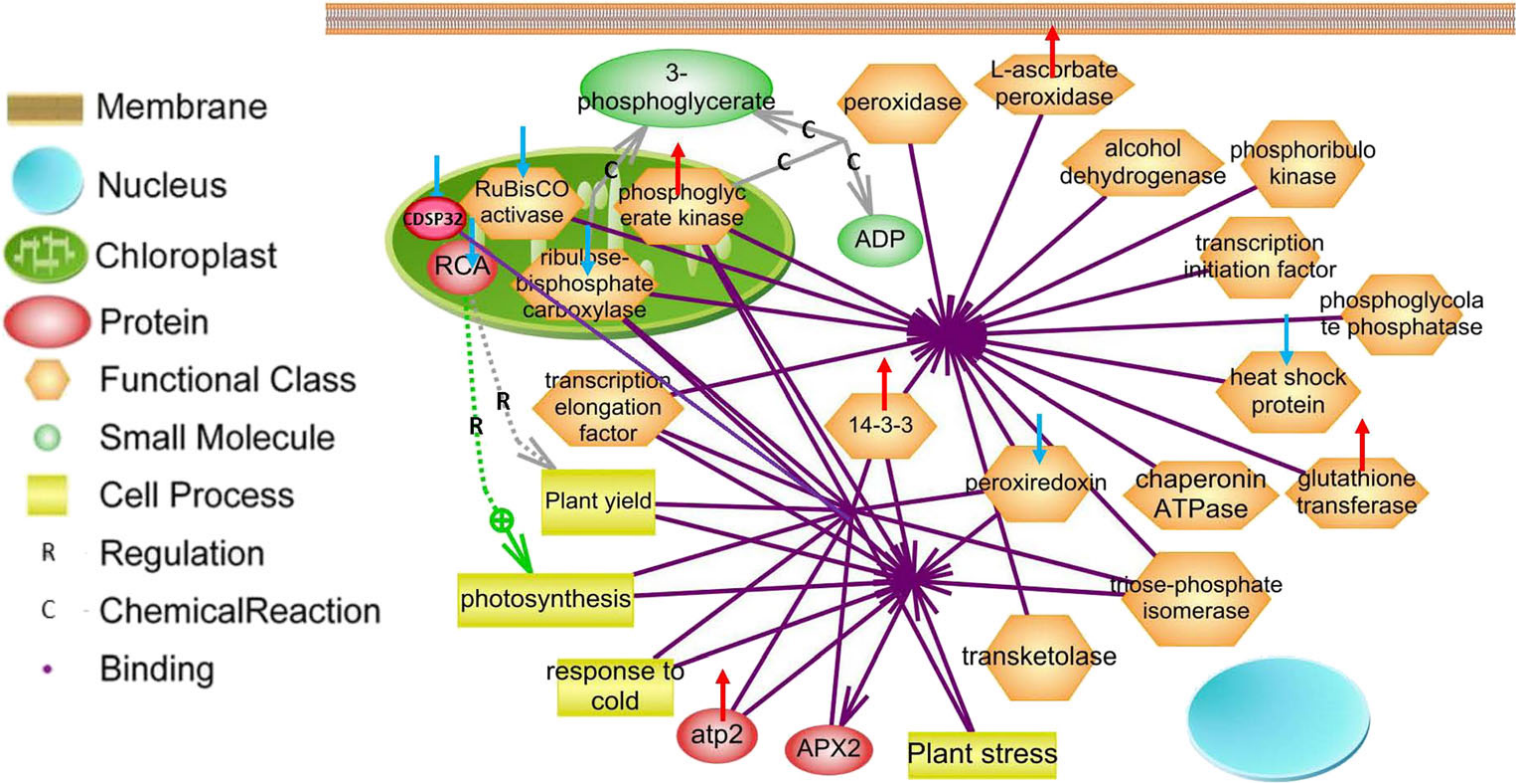
Biological networks generated for combination of 11 differential proteins. Differential proteins including ATP synthase subunit beta, RCA, Rubisco, phosphoglycerate kinase, APX, CDSP32, peroxiredoxin, chaperone, heat shock protein, glutathione transferase, and 14-3-3 were used to generate a protein-protein interaction network through Pathway Studio analysis. Regulation is marked as an *arrow with R*, chemical reaction as an *arrow with C*, and binding as an *arrow without any marks. Blue arrows* indicate down-regulated expression, and read indicates up-regulated expression (Color figure online)

All differential proteins identified were used to generate a wider protein interaction map by employing a Pathway Studio software program (Fig. 10). The relationships of binding, regulation, and chemical reaction were established for 11 differential proteins, responding to plant photosynthesis, yield, stresses, and cold. CDSP32 is localized in the chloroplast. There are direct interactions between 11 proteins, including ATP synthase subunit beta, Rubisco activase (RCA), Rubisco, phosphoglycerate kinase, APX, CDSP32, peroxiredoxin, chaperone, heat shock protein, glutathione transferase, and 14-3-3, whereas RCA can establish relations with other proteins through regulating the processes of photosynthesis and plant yield. The differential proteins including RCA and Rubisco activase, involved with photosynthetic biosynthesis, and CDSP32 and peroxiredoxin, related with stress, were decreased in both cassava genotypes under cold stress.

## Discussions

Cold stress adversely affects plant growth and development. Most temperate plants acquire cold tolerance by a process called cold acclimation (Chinnusamy et al. 2007). Low temperature has been regarded as a major stress for crops, and its negative effects have been studied extensively (Lyons 1973; Wettstein et al. 1995; Li et al. 2012). Proteomics was not only applied to describe the whole proteomes for the cell, tissue, organelle, and the total plants, but also used as a powerful tool for detecting the changes of the global proteins affected by the exposure to cold or other extreme environmental factors. Cassava proteomes could be identified using SDS-PAGE and 2-DE in combination with mass spectrometry and iTRAQ-based analysis, which have contributed to understanding the mechanism of storage root formation and the pathways associated with post-harvest physiological deterioration (Owiti et al. 2011; Batista de Souza et al. 2015). Comparative proteomic approaches were also applied to monitor differentially expressed leaf proteins during root transition from fibrous to tuberous, suggesting the possible metabolic switches in the leaf that may trigger/regulate storage root initiation and growth (Mitprasat et al. 2011). In the present study, we try to use proteomics techniques to understand the mechanisms that allow some plants to survive while others are sensitive under cold stress.

Low temperature could negatively influence crop productivity indirectly through its impact on depression of photosynthesis. Previous studies have shown that chlorophyll fluorescence measurement has been proven as an efficient and reproducible tool for evaluating plant susceptibility index to low temperature (Ehlert and Hincha 2008; Rizza et al. 2001). Plant cells can sense cold stress through low-temperature-induced changes in membrane fluidity and then initiate signal transduction to regulate the expression of genes involved in cold resistance responses (Vaultier et al. 2006). The present study showed that cold stress could induce the increase of EL and MDA (Fig. 2c, e), the most important physiological and biochemical indexes, resulting in the deformation of the structure of cell membrane. Low temperature also caused the changes of membrane lipid unsaturation and then led to adjustment of the membrane fluidity (Hodgson and Raison 1991). Besides cell membrane used as a sensor of cold stress, it is documented that the actin cytoskeleton was involved in response to low temperature (Aon et al. 2000; Sangwan et al. 2001; Orvar et al. 2000). Actin filaments are linked to the plasma membrane and may relate with signal transduction (Aon et al. 2000). Remodeling of the cytoskeleton is known to mediate cell responses to a variety of signals (Mathur et al. 1999). Actin is able to interact with a large number of different actin-binding proteins. In the present study, the abundance of actin-binding protein (spot 42) was dramatically increased under cold stress (Tables 1 and 2, Figs. 4 and 5), further implicating actin in the response to cold (Wang et al. 2009). Another protein that dramatically increased in abundance during cold stress is 14-3-3 protein (spot 23). In plants, 14-3-3 proteins are reported to play a significant role in regulating primary metabolism, signal transduction, and subcellular and defense reactions (Ferl 2004; Roberts 2003). They were also identified as drought-responsive proteins (Zhao et al. 2015). Through binding kinases and phosphatases and then regulating their activities, 14-3-3 proteins can be used as integral components of signal transduction pathways (Aitken 1995; Camoni et al. 1998; Sehnke et al. 2002). In the present study, the changes of material and energy metabolism may result from the signal transduction irritated by cold stress. As shown in Tables 1 and 2 and Figs. 4 and 5, 67 and 58 % changed proteins in SC8 and Col1046, respectively, were involved in material and energy metabolism (photosynthesis, carbohydrate and energy metabolism, protein biosynthesis, and amino acid metabolism). This suggests that altered metabolism seems to play an appreciable role in the cold response of cassava. Specifically, photosynthesis appears to be inhibited, as evidenced by the expressed decrease in photosynthetic apparatus proteins, such as seven down-regulated spots and three down-regulated spots in SC8 and nine down-regulated spots and two up-regulated spots in Co11046, inferring that the photosynthetic levels of both cassava plants were decreased. These data were confirmed by the analysis of SC8 and Co11046 chlorophyll a and chlorophyll b contents (Fig. 2a, b). These altered proteins might protect cassava leaves from photo-inhibition, which is typically aggravated when abiotic stresses are accompanied by light stress (Moon et al. 1995). In the present study, the expressions of Rubisco activase small chain SU26 (spot 20) and oxygen-evolving enhancer protein (spot 36) were increased. This result was similar as that shown under high salinity (Wang et al. 2008). The role of increased Rubisco levels in abiotic stress requires further clarification. In contrast to the apparent decrease in the expression of photosynthesis-related proteins, the activities of carbohydrate and energy metabolism-associated proteins were enhanced during the cold stress, including ATP synthase (spots 2 and 3), phosphoglycerate kinase (spot 18), and carbohydrate kinase FGGY (spot 21). All these proteins are enzymes of either the glycolysis or the TCA cycle pathways, which might ensure the energy flow as well as reductants to help cassava in resisting cold stress. The activities of protein biosynthesis-related proteins (three up-regulated spots, 1, 11, and 44; one down-regulated spot, 28) in SC8 genotype were increased under cold stress, suggesting a heightened production regarding protein synthesis. However, the activities of 5-methyltetrahydropteroyltriglutamate/homocysteine S-methyltransferase (spot 39) in both genotypes associated with amino acid metabolism were decreased under cold stress; its contribution to cassava cold resistance is still elusive.

Along with the above-mentioned components of signal transduction (spot 23), cold stress also up-regulated several proteins typically associated with defense, detoxifying, and antioxidant (spots 29, 34, and 43) (Wang et al. 2009). However, the heat shock proteins (HSPs) are an important class of defense proteins, which is a large and diverse group of molecular chaperones (Chen et al. 2009). Small HSPs were also supposed to act in association with other HSP like HSP70; they are particularly involved in stabilizing protein conformation by preventing the aggregation of denatured or incompletely folded proteins and by promoting the re-naturation of aggregated proteins (Boston et al. 1996). Stabilizing and refolding proteins with HSPs were reported as a major mechanism of cold resistance in plants (Thomashow 1998). HSP19 (spot 64), identified in Col1046, was previously described as developmental and stress response in tomato fruit (Neta-Sharir et al. 2005); CDSP32 (spot 19), a thioredoxin induced by extremely environmental stresses, is also a defense protein (Rey et al. 2005). Metacaspase (spot 43) was an up-regulated defense-related protein in the present study. It induced programmed cell death in plants and was arginine/lysine specific (Vercammen et al. 2004; Bozhkov et al. 2005).

Plants exposed to cold temperatures would generate reactive oxygen species (ROS). The previous studies reported that detoxifying-and-antioxidant-associated proteins, such as SOD and APX, were up-regulated to eliminate excess ROS under extremely environmental stresses (Sairam et al. 2005; Cavalcanti et al. 2007; Chen and Heuer 2013). SOD converts superoxide to the less toxic hydrogen peroxide (H_2_O_2_) molecule, and its function as an antioxidant has been reported in response to various abiotic stresses (Torabi et al. 2009; Parker et al. 2006; Gazanchian et al. 2007). The detoxification of H_2_O_2_ may be accomplished with antioxidant enzymes such as APX (spot 34) and 2-cys peroxiredoxin (spot 27), a member of a ubiquitous group of peroxidases that reduce H_2_O_2_ and alkyl hydroperoxide (Dietz et al. 2002). 2-Cys peroxiredoxin was down-regulated in SC8 and Col1046 under cold stress in the present study, suggesting that 2-cys peroxiredoxin-dependent water-water cycle would be an important alternative to detoxify H_2_O_2_ under oxidative stress (Hajheidari et al. 2005; Ali and Komatsu 2006; Hajheidari et al. 2007). Glutathione S-transferase (GST) (spot 29) was only up-regulated in SC8. It limits oxidative damage by removing ROS (Edwards et al. 2000). This may partly explain the reason that cassava SC8 has more cold-tolerant ability than Col1046, confirmed by the data analysis of EL and MDA content shown in Fig. 2c, d. Overall, the predominant changes in the expressions of global proteins related to oxidative stress defense underscore the importance of managing ROS and oxidative damage during cold acclimation and the protection of plant cells (Wang et al. 2009).

## Conclusions

In the present study, the dynamic expression changes of physiology and proteome in cassava genotypes SC8 and Col1046 have shown that cold-induced response would be an integrative process controlled by the complex biological networks involved in 64 differential proteins classified into 11 functions. Cassava cells might sense cold stress through low-temperature-induced responses in the plasma membrane and then transduce the cold stress signaling to plant defense system by 14-3-3 protein-mediated pathway. The whole defense system involved in the proteins associated with photosynthesis, carbohydrate and energy metabolism, defense, detoxifying and antioxidant, and signal transduction might elucidate the molecular mechanisms related to low temperature stress, reflecting that the trend is consistent with the physiological responses. These data should provide a useful clue to develop a complete understanding of how the plant cell responds and survives in the face of low temperatures.

## Electronic supplementary material

Below is the link to the electronic supplementary material.

Table S1 Correlation analysis of the indexes under low temperature. * indicated statistically significant differences at P<0.05, ** indicated statistically significant differences at P<0.01. (DOC 35 kb)

Table S2 Principal component analysis in cassava leaves under low temperature. The first PC was decided by REC, chlorophyll and MDA content; the second PC was consisting of activities of SOD and POD; the third PC was decided by free proline content. (DOC 30 kb)

## Abbreviations

APX: Ascorbate peroxidase
Rubisco: Ribulose-1,5-bisphosphate carboxylase
ATP: Adenosine-5′-triphosphate
EL: Electrolyte leakage
GST: Glutathione S-transferase
HSP: Heat shock protein
MALDI-TOF-TOF-MS: Matrix-assisted laser desorption ionization-time of flight mass spectrometry
MDA: Malondialdehyde
NBT: Nitro blue tetrazolium
PAGE: Polyacrylamide gel electrophoresis
POD: Peroxidase
RCA: Rubisco activase
ROS: Reactive oxygen species
SDS: Sodium dodecyl sulfate
SOD: Superoxide dismutase
TBA: Thiobarbituric acid
