# Supplementary material for: The Comparatively Proteomic Analysis in Response to Cold Stress in Cassava Plantlets
Source: Plant Mol Biol Report. 2016 May 6;34(6):1095–110. doi: 10.1007/s11105-016-0987-x (PMC5099363; doi:10.1007/s11105-016-0987-x)
Supplement: Supplementary file 1 — Correlation analysis of the indexes under low temperature. * indicated statistically significant differences at P<0.05, ** indicated statistically significant differences at P<0.01. (DOC 35 kb) [file 11105_2016_987_MOESM1_ESM.doc]

**Table S1**

| **Index** | **Chlorophyll** | **EL** | **Proline** | **MDA** | **Soluble sugar** | | **SOD**  **activity** | **POD activity** |
| --- | --- | --- | --- | --- | --- | --- | --- | --- |
| Chlorophyll | 1.000 |  |  |  |  | |  |  |
| EL | -0.721* | 1.000 |  |  |  | |  |  |
| Proline | -0.473 | 0.666* | 1.000 |  |  |  | |  |
| MDA | -0.641* | 0.721* | 0.172 | 1.000 |  |  | |  |
| Soluble sugar | -0.842** | 0.538 | 0.553 | 0.298 | 1.000 |  | |  |
| SOD activity | -0.045 | -0.409 | 0.258 | -0.611 | 0.292 | 1.000 | |  |
| POD activity | -0.200 | -0.342 | -0.044 | -0.297 | 0.188 | 0.713* | | 1.000 |
