# Supplementary material for: The Comparatively Proteomic Analysis in Response to Cold Stress in Cassava Plantlets
Source: Plant Mol Biol Report. 2016 May 6;34(6):1095–110. doi: 10.1007/s11105-016-0987-x (PMC5099363; doi:10.1007/s11105-016-0987-x)
Supplement: Supplementary file 2 — Principal component analysis in cassava leaves under low temperature. The first PC was decided by REC, chlorophyll and MDA content; the second PC was consisting of activities of SOD and POD; the third PC was decided by free proline content. (DOC 30 kb) [file 11105_2016_987_MOESM2_ESM.doc]

**Table S2**

| **Feature vector** | **Principal component** | | |
| --- | --- | --- | --- |
|  | 1 | 2 | 3 |
| EL | 0.515 | -0.129 | 0.171 |
| Chlorophyll | -0.485 | -0.206 | 0.339 |
| MDA | 0.419 | -0.289 | -0.405 |
| Soluble sugar | 0.409 | 0.344 | 0.053 |
| Proline | 0.361 | 0.232 | 0.664 |
| SOD activity | -0.131 | 0.627 | 0.149 |
| POD activity | -0.101 | 0.539 | -0.474 |
